# Effects of preoperative albumin-to-globulin ratio on overall survival and quality of life in esophageal cell squamous carcinoma patients: a prospective cohort study

**DOI:** 10.1186/s12885-023-10809-2

**Published:** 2023-04-13

**Authors:** Juwei Zhang, Zheng Lin, Jinsong Zhou, Yue Huang, Siting Chen, Yuan Deng, Minglian Qiu, Yuanmei Chen, Zhijian Hu

**Affiliations:** 1 Department of Epidemiology and Health Statistics, Fujian Provincial Key Laboratory of Environment Factors and Cancer, School of Public Health, Key Laboratory of Ministry of Education for Gastrointestinal Cancer, Fujian Medical University, Fuzhou, 350122 China; 2 Department of Thoracic Surgery, The First Affiliated Hospital of Fujian Medical University, Fuzhou, 350004 China; 3 Department of Thoracic Surgery, Fujian Provincial Cancer Hospital Affiliation to Fujian Medical University, Fuzhou, 350014 China; 4 Key Laboratory of the Ministry of Education for Gastrointestinal Cancer, Fujian Medical University, Fuzhou, 350122 China

**Keywords:** Esophageal squamous cell carcinoma, Albumin-to-globulin ratio, Overall survival, Health-related quality of life, Time to deterioration model

## Abstract

**Objective:**

This study aimed to investigate the effect of preoperative albumin-to-globulin ratio (AGR) on overall survival (OS) and health-related quality of life in patients with esophageal cell squamous carcinoma (ESCC).

**Methods:**

Serum albumin and globulin were measured within one week before surgery. Multiple follow-ups were conducted among patients with ESCC in the study in order to assess their life quality. The method used in the study was a telephone interview. Quality of life was measured using the EORTC Quality of Life Questionnaire-Core Questionnaire (EORTC QLQ-C30, version 3.0) and Esophageal Cancer Module (EORTC QLQ- OES18).

**Results:**

A total of 571 ESCC patients were included in the study. The results illustrated that 5-year OS of high AGR group (74.3%) was better than the low one (62.3%) (*P* = 0.0068). Univariate and multivariate Cox regression analysis found that preoperative AGR (*HR* = 0.642, *95%CI*: 0.444–0.927) are prognostic factor for patients with ESCC after surgery. In terms of quality of life, found that low AGR associated with increased postoperative time to deterioration (TTD) events in ESCC patients, and compared to low AGR, high AGR could delay the deterioration of emotional functioning(*P* = 0.001), dysphagia(*P* = 0.033), trouble with taste(*P* = 0.043) and speech problems(*P* = 0.043). After using the multivariate Cox regression analysis showed that high AGR could improve patients’ emotional function (*HR* = 0.657, *95% CI*: 0.507–0.852) and trouble with taste (*HR* = 0.706, *95% CI*: 0.514–0.971).

**Conclusions:**

Preoperative AGR in patients with ESCC after esophagectomy was positively correlated with overall survival rate and quality of life after operation.

**Supplementary Information:**

The online version contains supplementary material available at 10.1186/s12885-023-10809-2.

## Introduction

Esophageal cancer (EC) is a common upper gastrointestinal tumor, and its main histopathological types are squamous cell carcinoma (SCC) and adenocarcinoma (AC). According to the 2020 global cancer statistics, the incidence and mortality of esophageal cancer rank seventh and sixth in the world, respectively [1]. In China, 90% of esophageal cancer patients are squamous cell carcinoma, with male morbidity are about twice than women [2]. Despite radical esophagectomy combined with adjuvant therapy can improve patient survival, these treatments may cause adverse events in some patients, such as dysphagia, nausea, and vomiting [3].

Health-related quality of life can measure physical, mental, and social dimensions of health, as well as the physical or psychological impact of disease and treatment [4]. With the advancement of cancer diagnosis and treatment technology, the survival of cancer patients has been improved greatly [5]. At this time, many researchers have also realized the importance of improving the quality of life of cancer patients. Hence, it is crucial that find ways to improve esophageal squamous cell carcinoma (ESCC) patient prognosis and reduce postoperative adverse events.

Systemic inflammation and nutritional status are correlated with survival in cancer patients [6]. Albumin and globulin are two major components of human serum protein, which can reflect nutritional and inflammatory status of human bodies [7]. AGR better reflects the nutritional and inflammatory state by combining these two indicators in one measure [8]. Preoperative AGR has also been shown to be associated with postoperative survival in cancer patients [9]. In recent years, studies on the association between low preoperative AGR is related to unfavorable prognosis in patients with esophageal squamous cell carcinoma have also been reported [10].

**In terms of quality of life, more research is on the association between albumin and quality of life.** Han et al. revealed that lower serum albumin level was associated with impaired health-related quality of life in centenarians [11]. Balderas-Peña et al. demonstrated that a positive correlation between serum albumin and physical, emotional and social functioning scales in colorectal cancer patients [12]. These studies implicate that high level of serum albumin can improve some functions and symptoms in cancer patients. However, there are no reports on the relationship between preoperative AGR and postoperative quality of life in cancer patients. Specifically, the effects of preoperative AGR on quality of life in ESCC patients still remains unclear.

In the present study, we assessed the prognostic value of preoperative AGR and explored its impact on the quality of life of ESCC patients. This is the first study to investigate the effect of preoperative AGR on the postoperative quality of life of ESCC patients, which has important guiding significance for clinicians to treat cancer patients according to the preoperative conditions.

## Methods

### Study design and participants

This study was conducted among patients of in the First Affiliated Hospital of Fujian Medical University and Fujian Cancer Hospital from December 2014 to July 2021. Patients diagnosed with ESCC were invited to participate in the study in order to assess their life quality.

The inclusion criteria are outlined below: (1) patients with radical esophagectomy, (2) ESCC diagnosed by postoperative pathology, and (3) with clear TNM staging, (4) no preoperative chemotherapy and radiotherapy. Patients were excluded by the following criteria: (1) patients with other cancers, (2) patients with metastatic tumors or recurrent cases of esophageal cancer, (3) patients with incomplete clinical case information. The tumor stage was determined to be following the American Joint Committee on Cancer Tumor Lymph Node Metastasis (TNM) staging criteria. This study was approved by the Ethics Committee of Fujian Medical University (approval number: 201,495). Prior informed consent was obtained from all participants.

### Data collection

The demographics and clinical characteristics were collected from electronic medical records (EMR), including age, gender, tumor location, differentiation, T stage, lymph node status, radiochemotherapy, and TNM stage. Albumin and globulin counts were obtained by blood routine indexes one week before surgery, the ratios were calculated, and patients were grouped by median AGR (1.43) [13], high AGR group (≥ 1.43) and low AGR group (< 1.43).

All enrolled patients were interviewed face-to-face by trained interviewers using standardized questionnaire, which contains EORTC Quality of Life Questionnaire-Core Questionnaire (EORTC QLQ-C30, version 3.0) [14] and Esophageal Cancer Module (EORTC QLQ- OES18) [15] within 3 days of hospitalization. The EORTC QLQ-C30 scale is commonly used to assess quality of life in cancer patients, including global health status/quality of life (QOL) scale, five functional domains (physical, role, emotional, cognitive, and social functioning), and three symptom domains (fatigue, nausea/vomiting, pain) ), six single items (dyspnea, insomnia, loss of appetite, constipation, diarrhea, economic hardship) [16]. The EORTC QLQ-OES18 scale is an esophageal cancer-specific scale, includes four symptom areas (dysphagia, eating problems, reflux, pain swallowing) and six single items (swallowing saliva, choking while swallowing, dry mouth, taste problems, cough, and speech problems) [17]. Patients’ scores on the scale were converted to 0-100 according to the EORTC Scoring Manual (version 3.0). Higher scores in global health status and functional domains indicate better quality of life, while higher scores in the symptom domain show more severe symptoms [18].

### Follow-up and Health-Related Quality of Life Assessment

For postoperative patients, another group of trained interviewers based on telephone follow-up, starting after surgery, HRQOL was assessed every 3 months for the first year and then every six months. Survival time was defined as the time from surgery to death or the end of follow-up.

The time to deterioration (TTD) model was used to evaluate quality of life scale, including the time to deterioration and the number of deterioration events in each domain. In this study, time to deterioration was defined as the time from the start of the study to the first 5-point drop in the quality of life score compared to the baseline score, and if the patient did not deteriorate, it was reviewed at the time of the last completed quality of life [19].

### Statistical methods

Categorical variables were compared between different groups using the chi-squared test or Fisher’s exact test when necessary. Non-normally distributed data were expressed as median and interquartile range, and were compared using the non-parametric Mann-Whitney U test. Survival curves were generated using the Kaplan-Meier method, and survival and time to deterioration distributions were compared using the log-rank test. Univariate and multivariate Cox regression models were used to screen for factors associated with OS and quality of life in patients with ESCC. SPSS 22.0 was used to complete the statistical analysis described above. TTD model was constructed by the QoLR package of the R software to calculate scores on the EORTC QLQ-C30/EORTC QLQ-OES18 scale. 95% confidence interval (95% CI) is used to estimate hazard ratios (HR). All statistical tests were two-sided with a significance level of 5%, and *P* < 0.05 was considered statistically significant.

## Results

### Association with clinical and pathologic features

The clinicopathologic features of 571 patients with ESCC are stratified by preoperative AGR level and summarized in Table 1. The median follow-up of 38 months (range, 3–78 months). There were no statistically significant differences in tumor location, T stage, lymph node status, radiochemotherapy, and TNM stage between the low and normal preoperative AGR groups. The proportion of patients in the low AGR group with female (30%), high age (52.1%) and moderate differentiation (74.8%) were higher than that in the high AGR group.

**Table 1:** Clinicopathologic features of 571 patients with ESCC, stratified by preoperative serum albumin-to-globulin ratio (AGR).

Variables	AGR < 1.43 [n (%)]	AGR ≥ 1.43 [n (%)]	*χ* ^*2*^	*P* value
Sex			8.297	0.004
Female	86 (30.0)	56 (19.7)		
Male	200 (70.0)	229 (80.3)		
Age (years)			5.324	0.021
<61	137 (47.9)	164 (57.5)		
≥61	149 (52.1)	121 (42.5)		
Tumor location			0.242	0.886
Upper	41 (14.3)	42 (14.7)		
Middle	139 (48.6)	143 (50.2)		
Lower	106 (37.1)	100 (35.1)		
Differentiation			10.226	0.006
Well	23 (8.0)	24 (8.4)		
Moderate	214 (74.8)	181 (63.5)		
Poor/Undifferentiated	49 (17.1)	80 (28.1)		
T stage			1.776	0.183
T1-T2	98 (34.3)	113 (39.7)		
T3-T4	188 (65.7)	172 (60.4)		
Lymph node status			1.912	0.167
Negative	139 (48.6)	155 (54.4)		
Positive	147 (51.4)	130(45.60)		
Radiochemotherapy			0.673	0.412
No	167 (58.4)	176 (61.8)		
Yes	119 (41.6)	109 (38.3)		
TNM stage			0.784	0.376
I-II	150 (52.5)	160 (56.1)		
III-IV	136 (47.6)	125 (43.9)		

### Prognostic value of preoperative AGR in patients with ESCC

**Fig. 1:**
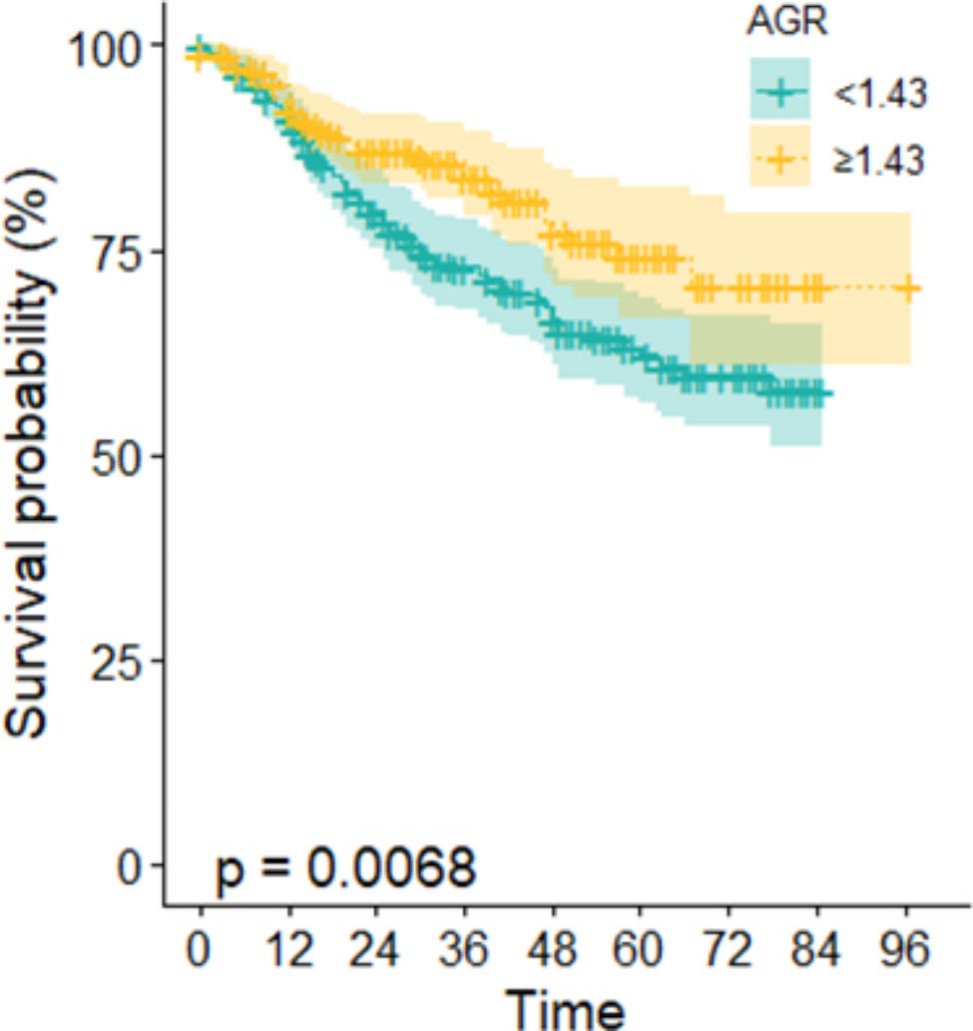
Kaplan-Meier survival curves of 571 esophageal squamous cell carcinoma patients

During follow-up, 137 (24.1%) patients died, 44 (15%) patients with high AGR and 93 (32.5%) patients with low AGR. Kaplan-Meier method indicated that the 5-year survival rate was 67.30% (95% CI: 0.625–0.725). The results of the Kaplan–Meier survival curves showed a significant difference in OS between patients with low and high serum AGR (P = 0.0068) (Fig. 1). Univariate analyses for OS showed that the AGR was a significant prognostic factor, as were radiochemotherapy and TNM stage. On multivariate analysis, after adjust sex, age, tumor location, differentiation, radiochemotherapy, and TNM stage, preoperative AGR remained independently correlated with OS, after adjustment for the effect of established clinical and pathological confounders (*HR* = 0.642, *95% CI*: 0.444–0.927, *P* = 0.018) (Table 2).

**Table 2:** Univariate and multivariate Cox proportional hazards analysis of clinicopathological factors for overall survival in patients who underwent curative surgery for ESCC.

Variables	Univariate			Multivariate	
	*HR (95%CI)*	*P* value		*HR (95%CI)* *	*P* value
Sex	1.215 (0.818–1.806)	0.334		-	-
Age	1.153 (0.825–1.612)	0.405		-	-
Tumor location	0.858 (0.667–1.103)	0.232		-	-
Differentiation	0.992 (0.724–1.359)	0.959		-	-
Radiochemotherapy	1.505 (1.077–2.104)	0.017		1.125 (0.790–1.601)	0.514
TNM stage	2.982 (2.084–4.266)	< 0.001		2.881 (1.979–4.194)	< 0.001
AGR	0.610 (0.425–0.877)	< 0.001		0.642 (0.444–0.927)	0.018

* Adjusted for sex, age, tumor location, differentiation, radiochemotherapy, and TNM stage

### Follow-up results

414 of 571 patients had quality of life information. Baseline characteristics of patients included (n = 414) and excluded (n = 157) in the present study were overall comparable (*P* > 0.05) (See supplement Table 1). The Baseline demographic and clinical characteristics of 414 ESCC patients with low and high AGR showed in Supplement Table 2. The results showed that the distribution of patients in the two groups was uneven in gender, age, and differentiation. Therefore, the patients were stratified according to gender, age, and differentiation. The results showed that high AGR could improve the emotional function of patients in the male, elderly, and poor or undifferentiated groups, while no significant improvement was observed in other layers (See supplement Fig. 1**)**. Supplement Table 3 presented the results of follow-up.

**Table 3:** The incidence of TTD events in each dimension of the QLQ-C30/ QLQ-OES18 scale in ESCC patients with low and high AGR

Domain/scale	AGR < 1.43 [n (%)]	AGR ≥ 1.43 [n (%)]	*χ* ^*2*^	*P* value
QLQ-C30				
Global health status/QOL	151 (75.1)	151 (70.9)	0.939	0.333
Functional scales				
Physical functioning	156 (77.6)	168 (78.9)	0.097	0.756
Role functioning	141 (70.1)	137 (64.3)	1.593	0.207
Emotional functioning	144 (71.6)	102 (47.9)	24.200	< 0.001
Cognitive functioning	123 (61.2)	88 (41.3)	16.353	< 0.001
Social functioning	141 (70.1)	127 (59.6)	5.018	0.025
Symptom scales				
Fatigue	144 (71.6)	125 (58.7)	7.628	0.006
Nausea/vomiting	136 (67.7)	104 (48.4)	15.058	< 0.001
Pain	129 (64.2)	103 (48.4)	10.509	0.001
Dyspnea	124 (61.7)	98 (46.0)	10.227	0.001
Insomnia	128 (63.7)	103 (48.4)	9.847	0.002
Appetite loss	135 (67.2)	104 (48.8)	14.251	< 0.001
Constipation	94 (46.8)	68 (31.9)	9.563	0.002
Diarrhea	125 (62.2)	100 (46.9)	9.681	0.002
QLQ-QES18				
General symptom scales				
Dysphagia	163 (81.1)	139 (65.3)	13.142	< 0.001
Eating problems	143 (71.1)	116 (54.5)	12.290	< 0.001
Reflux	168 (83.6)	148 (69.5)	11.377	0.001
Odynophagia	119 (59.2)	97 (45.5)	7.738	0.005
General symptom items				
Trouble swallowing saliva	109 (54.2)	79 (37.1)	12.255	< 0.001
Choking when swallowing	113 (56.2)	101 (47.4)	3.208	0.073
Dry mouth	103 (51.2)	88 (41.3)	4.103	0.043
Trouble with taste	101 (50.2)	66 (31.0)	15.944	< 0.001
Coughing	95 (47.3)	74 (34.7)	6.712	0.010
Speech problems	110 (54.7)	80 (37.6)	12.274	< 0.001

### Baseline quality-of-life scores

Median and quartiles were used to describe baseline quality of life scores. Baseline scores were statistically different between the two groups in the domains of global health status, physical function, dyspnea, constipation, eating problems, reflux, choking when swallowing, trouble with taste, coughing and speech problems(*P* < 0.05), no differences in other domains(*P* > 0.05) (Supplement Table 4).

**Table 4:** Determination of clinically meaningful time to deterioration in the EORTC QLQ -C30/EORTC QLQ -OES18 scale in ESCC patients with low and high AGR

		Time to deterioration [M (95%CI)], n = 414						
Domain/scale		AGR < 1.43		AGR ≥ 1.43		*χ* ^2^	*P *value	
**QLQ-C30**								
Global health status/QOL		14.029(11.431–16.626)		12.189(9.810-14.568)		0.725		0.395
Functional scales								
Physical functioning		14.029(11.655–16.403)		12.222(10.892–13.552)		3.414		0.065
Role functioning		18.891(14.131–23.651)		15.047(11.440-18.654)		1.070		0.301
Emotional functioning		15.803(13.685–17.920)		28.780(14.221–43.340)		10.927		0.001
Cognitive functioning		40.115(26.296–53.934)		48.493(35.090-61.896)		0.969		0.325
Social functioning		21.947(14.635–29.259)		20.041(14.018–26.064)		1.807		0.179
Symptom scales								
Fatigue		16.164(13.896–18.433)		19.680(14.538–24.821)		1.728		0.189
Nausea/vomiting		23.458(18.052–28.863)		31.014(25.128–36.901)		1.606		0.205
Pain		20.140(14.314–25.966)		34.563(25.072–44.053)		3.047		0.081
Dyspnea		25.692(19.114–32.270)		34.563(24.273–44.852)		1.261		0.261
Insomnia		24.674(19.438–29.909)		31.934(24.260-39.609)		1.353		0.245
Appetite loss		21.125(15.376–26.875)		35.713(19.997–51.428)		3.409		0.065
Constipation		46.259(26.890-65.628)		48.887(35.165–62.609)		1.212		0.271
Diarrhea		25.692(21.199–30.185)		32.657(23.040-42.174)		0.694		0.405
**QLQ-QES18**								
General symptom scales								
Dysphagia		12.977(11.279–14.676)		15.573(13.854–17.292)		4.563		0.033
Eating problems		16.033(13.697–18.369)		20.041(13.085–26.997)		1.715		0.190
Reflux		15.803(13.644–17.691)		14.916(12.235–17.597)		0.158		0.691
Odynophagia		26.021(15.968–36.073)		31.474(22.637–40.312)		0.362		0.547
General symptom items								
Trouble swallowing saliva		37.684(28.762–46.606)		42.349(33.760-50.938)		1.399		0.237
Choking when swallowing		29.536(25.282–33.789)		33.413(18.031–48.795)		0.210		0.647
Dry mouth		47.343(32.649–62.036)		48.099(31.596–63.601)		0.054		0.817
Trouble with taste		45.864(28.196–63.533)		66.300(49.393–83.206)		4.093		0.043
Coughing		47.934(31.196–64.672)		56.969(NA)		0.387		0.534
Speech problems		26.021(15.559–36.482)		48.099(33.274–62.924)		4.103		0.043

### Low AGR associated with increased postoperative TTD events in ESCC patients

The number of quality-of-life deterioration events was unevenly distributed between the high and low AGR groups. Compared to high AGR group, emotional function (*P* < 0.001), cognitive function (*P* < 0.001), social functioning (*P* = 0.025), fatigue (*P* = 0.006), nausea/vomiting (*P* < 0.001), pain (*P* = 0.001), dyspnea (*P* = 0.001), insomnia (*P* = 0.002), appetite loss (*P* < 0.001), constipation (*P* = 0.002), diarrhea (*P* = 0.002), dysphagia (*P* < 0.001), eating problems (*P* < 0.001), reflux, (*P* = 0.001) odynophagia (*P* = 0.005), trouble swallowing saliva (*P* < 0.001), dry mouth (*P* = 0.043), trouble with taste (*P* < 0.001), cough (*P* = 0.010), and speech problems (*P* < 0.001) had higher incidences of deterioration in patients with low AGR (Table 3).

### High preoperative AGR had a positive effect on quality of life of postoperative patients with ESCC

Log-rank test was used to analyze the time to deterioration in the EORTC QLQ -C30/EORTC QLQ -OES18 scale in ESCC patients with low and high AGR, compared to low AGR, high AGR could delay the deterioration of emotional functioning(*P* = 0.001), dysphagia(*P* = 0.033), trouble with taste(*P* = 0.043) and speech problems(*P* = 0.043) (Table 4).

After adjusting for sex, age, tumor location, differentiation, radiochemotherapy, and TNM stage, multivariate Cox regression analysis found that preoperative high AGR has a positive effect on the emotional function (*HR* = 0.657, *95% CI*: 0.507–0.852) and trouble with taste (*HR* = 0.706, *95% CI*: 0.514–0.971) of ESCC patients after surgery (Table 5).

**Table 5:** Association between preoperative AGR and EORTC QLQ-C30/EORTC QLQ-OES18 scale in ESCC patients

Domain/scale	Univariate			Multivariate	
	*HR* (95*%CI*)	*P* value		*HR* (95*%CI*) ^*^	*P* value
QLQ-C30					
Global health status/QOL	1.103 (0.880–1.383)	0.395		1.120 (0.889–1.411)	0.337
Physical functioning	1.229 (0.987–1.531)	0.065		1.211 (0.970–1.514)	0.091
Role functioning	1.133 (0.894–1.435)	0.302		1.160 (0.912–1.475)	0.226
Emotional functioning	0.653 (0.506–0.842)	0.001		0.657 (0.507–0.852)	0.002
Cognitive functioning	0.871 (0.661–1.148)	0.326		0.861 (0.650–1.140)	0.296
Social functioning	1.182 (0.926–1.508)	0.180		1.193 (0.931–1.529)	0.162
Fatigue	0.851 (0.670–1.083)	0.189		0.844 (0.661–1.077)	0.173
Nausea/vomiting	0.847 (0.654–1.096)	0.206		0.886 (0.682–1.150)	0.364
Pain	0.793 (0.611–1.030)	0.082		0.788 (0.604–1.027)	0.078
Dyspnea	0.858 (0.657–1.121)	0.262		0.876 (0.667–1.150)	0.340
Insomnia	0.857 (0.660–1.112)	0.246		0.851 (0.653–1.109)	0.232
Appetite loss	0.786 (0.608–1.016)	0.066		0.788 (0.608–1.022)	0.072
Constipation	0.838 (0.612–1.148)	0.272		0.826 (0.600-1.138)	0.243
Diarrhea	0.894 (0.687–1.164)	0.405		0.870 (0.665–1.139)	0.312
QLQ-QES18					
Dysphagia	0.782 (0.623–0.981)	0.033		0.803 (0.639–1.010)	0.061
Eating problems	0.849 (0.664–1.085)	0.191		0.858 (0.668-1.100)	0.227
Reflux	0.956 (0.766–1.193)	0.691		0.959 (0.764–1.204)	0.718
Odynophagia	0.920 (0.702–1.206)	0.548		0.888 (0.674–1.170)	0.398
Trouble swallowing saliva	0.838 (0.626–1.123)	0.238		0.804 (0.597–1.083)	0.151
Choking when swallowing	1.065 (0.813–1.396)	0.647		1.049 (0.797–1.380)	0.734
Dry mouth	1.035 (0.776–1.379)	0.817		1.020 (0.760–1.369)	0.897
Trouble with taste	0.725 (0.531–0.991)	0.044		0.706 (0.514–0.971)	0.032
Coughing	0.907 (0.668–1.233)	0.534		0.914 (0.669–1.248)	0.571
Speech problems	0.742 (0.555–0.992)	0.044		0.746 (0.556–1.002)	0.051

* Adjusting for sex, age, tumor location, differentiation, radiochemotherapy, and TNM stage

## Discussion

In recent years, the survival rate of cancer patients has been greatly improved, and more attention has shifted to improving the quality of life during survival. The present study revealed that ESCC patients with low AGR preoperatively had a poor OS postoperatively, the result was consistent with previous studies [20]. More importantly, we conducted about 6.5 years follow-ups for postoperative patients with ESCC, and the TTD model was used to calculate the time to deterioration and the number of deterioration events in each domain of the patient’s quality of life scale. Our study found that high preoperative AGR reduced TTD events and delayed deterioration of emotional functioning, dysphagia, trouble with taste, and speech problems. Meanwhile, multivariate Cox regression demonstrated that preoperative high AGR could improve emotional function and trouble with taste in postoperative patients with ESCC. These results implies that preoperative AGR can be used as a biomarker for the prognosis of ESCC patients and may have a positive effect on patient survival and quality of life improvement if preoperative intervention is performed.

Early studies reported that some EC patients would experience nausea, vomiting and loss of appetite after postoperative radiotherapy and chemotherapy, resulting in weight loss, and severe malnutrition [21]. Cancer patients with malnutrition were prone to infection, delayed recovery, and increased mortality after surgery, which is not conducive to prognosis [22]. Therefore, the physical state before surgery plays a key role in the effect of postoperative treatment. Serum albumin and globulin reflect human nutritional and inflammatory status, respectively [23]. Systemic inflammatory response and malnutrition are considered being important factors affecting the prognosis of patients with malignant tumors. Inflammatory cells and inflammatory mediators such as serotonin [24], interleukin (IL)-1 [25], and IL-6 [26]produced by the inflammatory response can promote tumor proliferation, metastasis, and invasion, these changes correlate with less favorable outcome. Conversely, reduce inflammation and increase nutrition may help improve outcomes [27]. Patients with gastrointestinal tumors were prone to malnutrition and result a bad prognosis [28]. while proper nutritional support before cancer treatment and nutritional monitoring during treatment contribute to improved quality of life and better clinical outcomes [29]. Therefore, preoperative nutritional supplementation is of great significance to improve the quality of life and prognosis of patients with gastrointestinal tumors. In addition, several studies also proved that lower preoperative AGR has a negative correlation to prognosis in gastric cancer [30], bladder cancer [31], and colorectal cancer [32], which consistent with our results.

High morbidity, mortality, and complication rates predispose patients with esophageal cancer to emotional distress and psychiatric disorders [33]. Meanwhile, a nationwide population-based longitudinal study showed that the number of EC patients incident psychological distress after surgery increased year by year [34]. In addition, negative emotions such as depression and anxiety may hamper cancer treatment and recovery, as well as quality of life and survival [35]. Therefore, improving the emotion of cancer patients after surgery is particularly important for the patient’s recovery. This study found that high preoperative AGR was positively associated with postoperative improvement in emotional function in ESCC patients. Decreased AGR level means low albumin or high globulin. Elevated globulin indicates an inflammatory response in the body. Acute and chronic inflammation can cause hypoalbuminemia through protein metabolism through inflammatory mechanisms, reducing albumin synthesis and inducing increased capillary permeability [36]. We speculate that cancer patients may lead to hypoalbuminemia through this mechanism, and thus are prone to adverse events such as decreased immunity and infection, and these changes can easily lead to negative emotions in patients. High AGR produces opposite clinical outcomes, as in this study. Our research results have important guiding significance for clinicians to make treatment plan decisions. Doctors obtain AGR information in a non-invasive manner before surgery and can determine whether measures such as anti-inflammatory, nutritional support, or correction of hypoproteinemia are necessary before performing surgery based on the level of AGR. In short, the results of preoperative AGR can indicate whether the patients need to improve their AGR levels first. Based on our results, we can know that high preoperative AGR levels have a positive impact on the quality of life of patients with esophageal squamous cell carcinoma after surgery, especially in terms of emotional function and trouble with taste. In subsequent clinical work, clinicians can evaluate the level of AGR before surgery. For people with low AGR, active measures such as correcting hypoproteinemia and nutritional support during the perioperative period, such as intravenous albumin infusion, intragastric or parenteral nutritional support, may have important clinical value in improving patients’ AGR status and anti-tumor immune function before surgery. Therefore, improve the patient’s preoperative physical condition through improving the AGR status, thereby improving the patient’s quality of life during survival, which reflects the important clinical significance of monitoring the preoperative AGR level on the impact of postoperative quality of life.

One advantage of this study is that the TTD model was used to evaluate the quality of life of patients longitudinally. The results of the TTD model are easy to be interpreted by clinicians, which is helpful for clinicians to observe factors affecting the quality of life of postoperative cancer patients before surgery to provide scientific guidance for patients. The long follow-up period is another strength. At the same time, this study also has some limitations. On the one hand, there is a possibility that follow-up bias may occur due to the long follow-up time in this study. on the other hand, this study only explored the association between preoperative AGR and the overall survival and quality of life of postoperative patients with ESCC. Subsequent studies can analyze the impact of postoperative AGR on patients.

In conclusion, our study found that high preoperative AGR was associated with better overall survival and emotional functioning, and less trouble with taste in patients with ESCC, which provides scientific guidance for clinicians to improve the anxiety and depressed emotions of patients.

## Electronic supplementary material

Below is the link to the electronic supplementary material.

Supplementary Material 1

Supplementary Material 2

## Data Availability

The datasets generated during and/or analysed during the current study are available from the corresponding author on reasonable request.

## Abbreviations

ESCC: Esophageal squamous cell carcinoma
EC: Esophageal cancer
SCC: Squamous cell carcinoma
AC: Adenocarcinoma
AGR: Albumin-to-globulin ratio
OS: Overall survival
HRQoL: Health-related quality of life
TTD: The time to deterioration
TNM: Tumor Lymph Node Metastasis
HR: Hazard ratio
95% CI: 95% confidence interval
