# Supplementary material for: Effects of preoperative albumin-to-globulin ratio on overall survival and quality of life in esophageal cell squamous carcinoma patients: a prospective cohort study
Source: BMC Cancer. 2023 Apr 13;23:342. doi: 10.1186/s12885-023-10809-2 (PMC10103440; doi:10.1186/s12885-023-10809-2)
Supplement: Supplementary file 1 — Supplementary Material 1 [file 12885_2023_10809_MOESM1_ESM.docx]

**Supplement table 1.** Baseline characteristics of patients between inclusion and exclusion.

| Variables | Inclusion [n (%)] | Exclusion [n (%)] | *χ^2^* | *P* value |
| --- | --- | --- | --- | --- |
| Sex |  |  | 0.769 | 0.381 |
| Female | 107 (25.8) | 35 (22.3) |  |  |
| Male | 307 (74.2) | 122 (77.7) |  |  |
| Age (years) |  |  | 0.002 | 0.964 |
| <61 | 218 (52.7) | 83 (52.9) |  |  |
| ≥61 | 196 (47.3) | 74 (47.1) |  |  |
| Tumor location |  |  | 5.135 | 0.077 |
| Upper | 61 (14.7) | 22 (14.0) |  |  |
| Middle | 193 (46.6) | 89 (56.7) |  |  |
| Lower | 160 (38.6) | 46 (29.3) |  |  |
| Differentiation |  |  | 4.136 | 0.126 |
| Well | 30 (7.2) | 17 (10.8) |  |  |
| Moderate | 296 (71.5) | 99 (63.1) |  |  |
| Poor/Undifferentiated | 88 (21.3) | 41 (26.1) |  |  |
| T stage |  |  | 0.608 | 0.436 |
| T1-T2 | 157 (37.9) | 54 (34.4) |  |  |
| T3-T4 | 257 (62.1) | 103 (65.6) |  |  |
| Lymph node status |  |  | 0.823 | 0.364 |
| Negative | 218 (52.7) | 76 (48.4) |  |  |
| Positive | 196 (47.3) | 81 (51.6) |  |  |
| Radiochemotherapy |  |  | 0.265 | 0.607 |
| No | 246 (59.4) | 97 (61.8) |  |  |
| Yes | 168 (40.6) | 60 (38.2) |  |  |
| TNM stage |  |  | 0.270 | 0.603 |
| Ⅰ-Ⅱ | 222 (53.6) | 88 (56.1) |  |  |
| Ⅲ-Ⅳ | 192 (46.4) | 69 (43.9) |  |  |
| AGR |  |  | 1.423 | 0.233 |
| AGR<1.43 | 201 (48.6) | 85 (54.1) |  |  |
| AGR≥1.43 | 213 (51.4) | 72 (45.9) |  |  |

**Supplement table 2.** Baseline demographic and clinical characteristics of ESCC patients.

| Variables | AGR<1.43 [n (%)] | AGR≥1.43 [n (%)] | *χ^2^* | *P* value |
| --- | --- | --- | --- | --- |
| Sex |  |  | 5.097 | 0.024 |
| Female | 62 (30.9) | 45 (21.1) |  |  |
| Male | 139 (69.2) | 168 (7.89) |  |  |
| Age (years) |  |  | 5.438 | 0.020 |
| <61 | 94 (46.8) | 124 (58.2) |  |  |
| ≥61 | 107 (53.2) | 89 (41.8) |  |  |
| Tumor location |  |  | 0.507 | 0.776 |
| Upper | 28 (13.9) | 33 (15.5) |  |  |
| Middle | 92 (45.8) | 101 (47.4) |  |  |
| Lower | 81 (40.3) | 79 (37.1) |  |  |
| Differentiation |  |  | 7.223 | 0.027 |
| Well | 12 (6.0) | 18 (8.5) |  |  |
| Moderate | 156 (77.6) | 140 (65.7) |  |  |
| Poor/Undifferentiated | 33 (16.4) | 55 (25.8) |  |  |
| T stage |  |  | 2.144 | 0.143 |
| T1-T2 | 69 (34.3) | 88 (41.3) |  |  |
| T3-T4 | 132 (65.7) | 125 (58.7) |  |  |
| Lymph node status |  |  | 0.909 | 0.340 |
| Negative | 101 (50.3) | 117 (54.9) |  |  |
| Positive | 100 (49.8) | 96 (45.1) |  |  |
| Chemotherapy |  |  | 0.238 | 0.626 |
| No | 117 (58.2) | 129 (60.6) |  |  |
| Yes | 84 (41.8) | 84 (39.4) |  |  |
| TNM stage |  |  | 0.301 | 0.583 |
| Ⅰ-Ⅱ | 105 (52.2) | 117 (54.9) |  |  |
| Ⅲ-Ⅳ | 96 (47.8) | 96 (45.1) |  |  |

**Supplement table 3.** Postoperative follow-up for ESCC patients.

| Follow-up times | Time to baseline follow-up (months) | Number of subjects | Follow-up rate |
| --- | --- | --- | --- |
| baseline | 0 | 414 | 100% |
| 1st | 3 | 227 | 72.76% |
| 2st | 6 | 241 | 77.24% |
| 3st | 9 | 228 | 81.14% |
| 4st | 12 | 184 | 83.64% |
| 5st | 18 | 166 | 86.01% |
| 6st | 24 | 124 | 84.93% |
| 7st | 30 | 117 | 86.67% |
| 8st | 36 | 114 | 89.73% |
| 9st | 42 | 100 | 92.59% |
| 10st | 48 | 88 | 96.70% |
| 11st | 54 | 82 | 100% |
| 12st | 60 | 66 | 100% |
| 13st | 66 | 47 | 100% |
| 14st | 72 | 14 | 100% |
| 15st | 78 | 7 | 100% |

**Supplement table 4.** Preoperative EORTC scale baseline scores in male post-ESCC patients.

|  |  | Baseline HRQOL scores [M (IQR)], n=414 | | | | | | |
| --- | --- | --- | --- | --- | --- | --- | --- | --- |
| Domain/scale |  | AGR<1.43 |  | AGR≥1.43 |  | Z | *P* value | |
| **QLQ-C30** |  |  |  |  |  |  |  |  |
| Global health status/QOL |  | 66.67(66.67, 83.33) |  | 83.33(66.67, 83.33) |  | -2.314 |  | 0.021 |
| Functional scales |  |  |  |  |  |  |  |  |
| Physical functioning |  | 100.00(86.67, 100.00) |  | 100.00(93.33, 100.00) |  | -2.095 |  | 0.036 |
| Role functioning |  | 100.00(100.00, 100.00) |  | 100.00(100.00, 100.00) |  | -0.573 |  | 0.567 |
| Emotional functioning |  | 91.67(83.33, 100.00) |  | 91.67(75.00, 100.00) |  | -1.812 |  | 0.070 |
| Cognitive functioning |  | 100.00(83.33, 100.00) |  | 100.00(100.00, 100.00) |  | -0.432 |  | 0.665 |
| Social functioning |  | 100.00(66.67, 100.00) |  | 83.33(66.67, 100.00) |  | -1.218 |  | 0.223 |
| Symptom scales |  |  |  |  |  |  |  |  |
| Fatigue |  | 0.00(0.00, 22.22) |  | 11.11(0.00, 22.22) |  | -0.590 |  | 0.555 |
| Nausea/vomiting |  | 0.00(0.00, 0.00) |  | 0.00(0.00, 0.00) |  | -1.663 |  | 0.096 |
| Pain |  | 0.00(0.00, 16.67) |  | 0.00(0.00, 16.67) |  | -0.937 |  | 0.349 |
| Dyspnea |  | 0.00(0.00, 0.00) |  | 0.00(0.00, 0.00) |  | -2.407 |  | 0.016 |
| Insomnia |  | 0.00(0.00, 33.33) |  | 0.00(0.00, 33.33) |  | -0.898 |  | 0.369 |
| Appetite loss |  | 0.00(0.00, 33.33) |  | 0.00(0.00, 0.00) |  | -1.268 |  | 0.205 |
| Constipation |  | 0.00(0.00, 0.00) |  | 0.00(0.00, 0.00) |  | -2.800 |  | 0.005 |
| Diarrhea |  | 0.00(0.00, 0.00) |  | 0.00(0.00, 0.00) |  | -0.715 |  | 0.475 |
| **QLQ-QES18** |  |  |  |  |  |  |  |  |
| General symptom scales |  |  |  |  |  |  |  |  |
| Dysphagia |  | 77.78(66.67, 77.78) |  | 88.89(66.67, 100.00) |  | -0.210 |  | 0.834 |
| Eating problems |  | 8.33(0.00, 16.67) |  | 0.00(0.00, 16.67) |  | -2.663 |  | 0.008 |
| Reflux |  | 0.00(0.00, 0.00) |  | 0.00(0.00, 0.00) |  | -2.109 |  | 0.035 |
| Odynophagia |  | 11.11(0.00, 22.22) |  | 11.11(0.00, 22.22) |  | -1.229 |  | 0.219 |
| General symptom items |  |  |  |  |  |  |  |  |
| Trouble swallowing saliva |  | 0.00(0.00, 0.00) |  | 0.00(0.00, 0.00) |  | -0.344 |  | 0.731 |
| Choking when swallowing |  | 33.33(0.00, 33.33) |  | 0.00(0.00, 33.33) |  | -2.963 |  | 0.003 |
| Dry mouth |  | 0.00(0.00, 33.33) |  | 0.00(0.00, 0.00) |  | -1.677 |  | 0.094 |
| Trouble with taste |  | 0.00(0.00, 0.00) |  | 0.00(0.00, 0.00) |  | -2.122 |  | 0.034 |
| Coughing |  | 0.00(0.00, 0.00) |  | 0.00(0.00, 0.00) |  | -2.068 |  | 0.039 |
| Speech problems |  | 0.00(0.00, 0.00) |  | 0.00(0.00, 0.00) |  | -1.964 |  | 0.050 |
