# Supplementary figures and images for: Effects of preoperative albumin-to-globulin ratio on overall survival and quality of life in esophageal cell squamous carcinoma patients: a prospective cohort study
Source: BMC Cancer. 2023 Apr 13;23:342. doi: 10.1186/s12885-023-10809-2 (PMC10103440; doi:10.1186/s12885-023-10809-2)

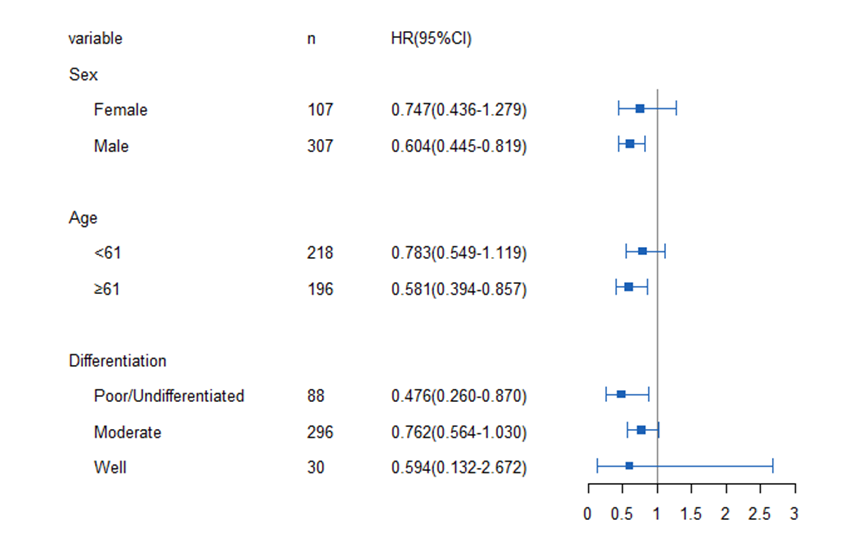


**Supplement figure 1.** Multivariate analysis results stratified by sex, age and differentiation.

Supplement: Supplementary file 2 — Supplementary Material 2 [file 12885_2023_10809_MOESM2_ESM.docx]
